# Independent modes of disease repair by AIM protein distinguished in AIM-felinized mice

**DOI:** 10.1038/s41598-018-31580-6

**Published:** 2018-09-03

**Authors:** Ryoichi Sugisawa, Ginga Komatsu, Emiri Hiramoto, Naoki Takeda, Ken-ichi Yamamura, Satoko Arai, Toru Miyazaki

**Affiliations:** 1 Laboratory of Molecular Biomedicine for Pathogenesis, Center for Disease Biology and Integrative Medicine, Faculty of Medicine, The University of Tokyo, Tokyo, 113-0033 Japan; 2 Center for Animal Resources and Development, Kumamoto University, Kumamoto, 860-0811 Japan; 3 AMED-CREST, Japan Agency for Medical Research and Development, Tokyo, 113-0033 Japan; 4 Max Planck-The University of Tokyo Center for Integrative Inflammology, Tokyo, 113-0033 Japan; 5 Present Address: School of Biochemistry and Immunology, Trinity Biomedical Sciences Institute, Trinity College Dublin, Dublin, Ireland

## Abstract

Tissue macrophage-derived apoptosis inhibitor of macrophage (AIM, encoded by *cd5l* gene) is a circulating protein that has suppressive functions in a broad range of diseases including obesity, liver steatosis, hepatocellular carcinoma (HCC), and acute kidney injury (AKI). In healthy states, high levels of AIM circulate in the inactivated state by associating with the immunoglobulin M (IgM) pentamer in the blood, whereas during AKI, AIM dissociates from IgM and gains disease repair activity. Here, we assessed whether AIM activation via its release from IgM is required to ameliorate other diseases. To this end, we employed a mouse line in which mouse AIM was replaced with feline AIM (AIM-felinized mice). Because feline AIM rarely dissociates from IgM due to its extremely high binding affinity for IgM, these mice exhibited deficient AKI repair as in cats. When fed a high-fat diet (HFD), similar to AIM-deficient (*AIM*^−/−^) mice, AIM-felinized mice exhibited enhanced triacylglycerol deposition in visceral adipocytes and hepatocytes, resulting in more prominent obesity and fatty liver than in wild-type mice. In contrast, the incidence of HCC after a 1-year HFD was remarkably lower in AIM-felinized mice than in *AIM*^−/−^ mice, suggesting that AIM produced by liver Kupffer macrophages might directly facilitate the elimination of HCC cells. Accordingly, the marked deposition of AIM accompanied by accumulation of Kupffer cells was obvious during HCC tumour development in AIM-felinized mice. Δsµ mice, which harbour almost no circulating AIM due to the lack of secreted IgM, showed a phenotype comparable with that of AIM-felinized mice in prevention of those diseases. Thus, blood AIM released from IgM contributes to suppression of obesity and fatty liver as in AKI, whereas macrophage-derived noncirculating AIM mainly prevents HCC development. Our study depicted two different modes of disease prevention/repair facilitated by AIM, which could be the basis for HCC therapy that works by increasing AIM expression in macrophages.

## Introduction

Apoptosis inhibitor of macrophage (AIM; encoded by *cd5l*)^1^ is a circulating protein that was initially identified as an apoptosis inhibitor supporting the survival of macrophages against various types of apoptosis-inducing stimuli^1^. It is produced by tissue-resident macrophages, mainly liver Kupffer cells, under transcriptional regulation by nuclear receptor liver X receptor/retinoid X receptor (LXR/RXR) heterodimers^2–4^. In the blood, AIM associates with immunoglobulin M (IgM) pentamers, which protect AIM from renal excretion, thereby maintaining high levels of circulating AIM (approximately 5 µg/mL in humans and 2 µg/mL in mice)^5–7^. Interestingly, in mice and humans with acute kidney injury (AKI), AIM dissociates from IgM pentamers and is excreted in the urine, although the mechanisms underlying AIM release from IgM remain unknown. We recently reported that IgM-free urinary AIM accumulates on AKI-associated intraluminal dead cell debris that obstructs renal proximal tubules and further exacerbates tubular injury^8^. AIM on the debris interacts with kidney injury molecule 1 (encoded by *havcr*1) expressed on injured tubular epithelial cells^8^, which promotes the phagocytic clearance of AIM-bound debris by epithelial cells^8–11^. Thus, IgM-free AIM contributes to recovery from AKI; namely, in healthy states, AIM remains inactive and accumulates in the blood though association with IgM, whereas it is released from IgM and achieves disease repair activity during AKI^8,12^.

The importance of AIM release from IgM in facilitating recovery from AKI was shown in cats, which are profoundly more susceptible than other animals to renal failure and death from the disease^13–16^. Due to a specific cluster of positively charged amino acids on the surface of feline AIM (which are not observed in mouse and human AIM), AIM associates with IgM approximately 1000-fold more strongly in cats than in mice; the dissociation constant, as determined by surface plasmon resonance, is 5.97 × 10^−9^ M in feline AIM/IgM-fragment crystallization (Fc) and 5.82 × 10^−6^ M in mouse AIM/IgM-Fc^17^. This causes no dissociation of AIM from IgM, and thus no AIM-excretion in the urine during AKI, resulting in insufficient repair of AKI^17^. This was confirmed by generating mice in which mouse AIM was replaced with feline AIM, thereby felinizing AIM in mice. As expected, IgM-free AIM is not detected during AKI induced by ischaemia/reperfusion in AIM-felinized mice, similar to observations in cats. Hence, efficient AKI repair is not facilitated in these mice, resulting in high mortality^17^. Thus, dissociation of AIM from the IgM pentamer in blood is an indispensable process for inducing the therapeutic effects of endogenous AIM for AKI.

In addition to AKI, accumulating evidence has shown that AIM also exhibits protective effects against various other diseases, particularly obesity^18^ and obesity-related fatty liver diseases including hepatocellular carcinoma (HCC)^19^. We demonstrated that AIM is incorporated into obese adipocytes and hepatocytes via cluster of differentiation 36 (CD36)-mediated endocytosis, where it inactivates cytoplasmic fatty acid synthase (FASN) through direct binding^18^. This response reduces the production of lipid droplet-coating proteins such as fat-specific protein 27 (FSP27) and perilipin, thereby decreasing triacylglycerol deposition within adipocytes and hepatocytes^18–20^. This results in the prevention of obesity and liver steatosis. Interestingly, however, unlike normal hepatocytes, HCC cells do not incorporate AIM; instead, AIM accumulates on their surface. AIM accumulation inactivates various regulators of complement activation on the surface of HCC cells, thereby provoking complement C3 deposition on the tumour cell surface, leading to necrotic cell death^19,21^. Accordingly, most AIM-deficient (*AIM*^−/−^) mice fed a high-fat diet (HFD) for 1 year developed HCC, whereas no wild-type (WT) mice developed the disease^19^. The increased incidence of HCC development was also observed when *AIM*^−/−^ mice were fed a high-fructose diet^22^.

We wondered whether the dissociation of AIM from the IgM pentamer in the blood is also required to regulate and prevent obesity, fatty liver, and HCC, similar to AKI. AIM-felinized mice were an ideal tool to address this question, as macrophages produce feline AIM under the same transcriptional regulation as endogenous mouse AIM, and AIM is not released from the IgM pentamer.

In this study, we investigated whether feline AIM is incorporated into adipocytes and hepatocytes and harbours lipolytic effects at levels comparable with that of mouse AIM. Then, we studied the progression of obesity, fatty liver, and associated inflammatory states in adipose tissues and the liver in mice fed an HFD. We also analysed the incidence of HCC after a long-term HFD challenge. The results showed two different modes of disease prevention by AIM.

## Results

### Feline AIM is incorporated into adipocytes and induces lipolysis

**Figure 1:**
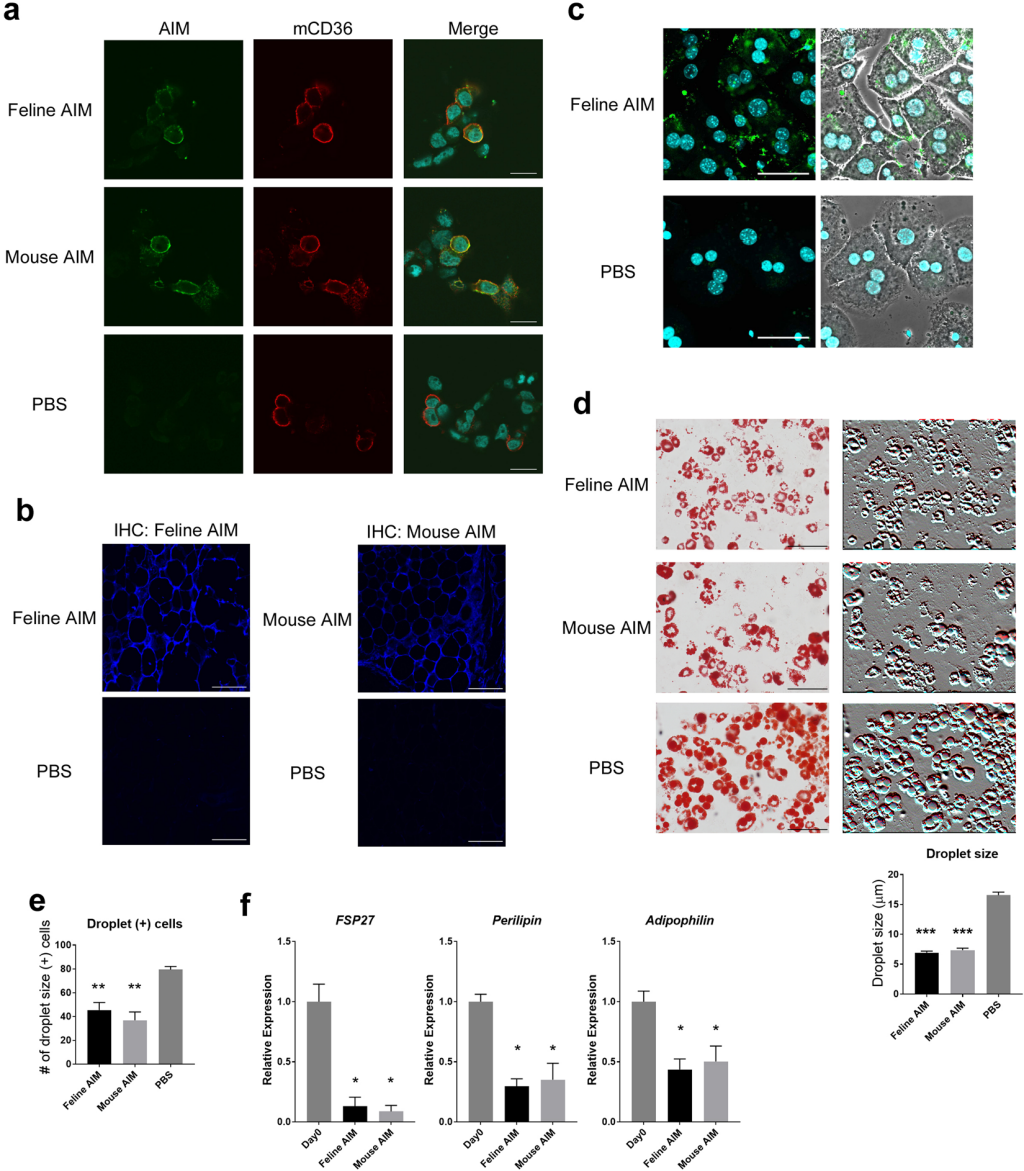
Comparable lipolytic function in feline and mouse AIM. **(a)** Representative photomicrographs of mCD36-expressing HEK293T cells stained for mCD36 (red) and mouse or feline AIM (green). Blue indicates Hoechst staining. Scale bars, 20 μm. **(b)** Uptake of rAIM by *AIM*^−/−^ adipocytes *in vivo*. Representative photomicrographs of epididymal fat stained for mouse or feline AIM (blue). *AIM* ^−/−^ mice were injected with mouse or feline rAIM or PBS directly into the epididymal fat (total 100 μg). Three hours after injection, tissue sections were obtained and stained for AIM. Scale bars, 100 μm. **(c)** Uptake of rAIM by AIM^−/−^ primary hepatocytes isolated from mouse liver. Primary hepatocytes were stained for rAIM (green) and nuclei (Hoechst). Phase contrast images are also presented at the right side. Scale bars, 50 μm. **(d,e)** Differentiated 3T3-L1 adipocytes (ins/DEX/IBMX stimulation+) were incubated with mouse or feline rAIM (10 μg/mL) or PBS for 6 days. **(d)** Representative photomicrographs of differentiated 3T3-L1 adipocytes showing staining for Oil Red O after rAIM or PBS treatment. Scale bars, 100 μm. Phase-contrast photo-images are also presented. Relative droplet size was assessed by evaluating the diameter of 50 droplets. Error bar indicates the standard error of the mean (SEM). **(e)** The numbers of droplet-containing cells are also shown (cells/microscopic field). Data are presented as the means of five independent areas. Error bar indicates the SEM. **(f)** 3T3-L1 adipocytes incubated with rAIM (20 µg/mL) for 0 or 2 days were analysed for mRNA levels of *FSP27*, *perilipin*, and *adipophilin* by qPCR. Values were normalized to those of glyceraldehyde 3-phosphate dehydrogenase (*GAPDH*) and presented as expression relative to that on day 0 of rAIM treatment. Error bar indicates the SEM.

To confirm that feline AIM is functionally equivalent to mouse AIM, we first assessed whether feline AIM enters cells via CD36-mediated endocytosis, which is the first step required for AIM to affect lipid storage within adipocytes and hepatocytes. To this end, FLAG-tagged mouse CD36 was overexpressed in HEK293T cells and treated with recombinant feline or mouse recombinant AIM (rAIM), after which AIM incorporation was determined by immunocytostaining. As shown in Fig. 1a, both feline and mouse AIM (green) were similarly endocytosed by CD36-expressing cells (red). Then, we injected both types of rAIM intravenously into *AIM*^−/−^ mice, and histologically examined the epididymal fat tissues for the internalized AIM protein. Again, immunohistochemical analysis clearly demonstrated that both feline and mouse AIM were well incorporated by adipocytes in the epididymal fat (Fig. 1b). We also addressed the entry of AIM into hepatocytes by treating primary hepatocytes isolated from the liver of *AIM*^−/−^ mice with feline or mouse rAIM, as histologic detection of intravenously injected AIM in the whole liver was not technically feasible. As demonstrated in Fig. 1c, incorporation of a level of feline rAIM by primary hepatocytes was observed.

We then compared the lipolytic impact of feline rAIM. To test this, differentiated 3T3-L1 adipocytes were challenged with feline rAIM or mouse rAIM as a control, in cell culture on day 6 of differentiation. The size of lipid droplets within the cells was reduced after the 6-day incubation of cells with feline and mouse rAIM (Fig. 1d). In addition, the number of cells containing lipid droplets was also decreased at similar levels in feline- or mouse-AIM treated cells (Fig. 1e). We previously demonstrated that incorporated AIM inactivates FASN, which results in reduction of the mRNA levels of *FSP27, perilipin*, and *adipophilin*, important elements involved in the formation of lipid droplets^23,24^, thereby promoting lipolysis^18,20^. As shown in Fig. 1f, mRNA levels of those three genes were decreased by treatment of 3T3-L1 adipocytes with feline or mouse AIM, and the effects of both types of AIMs were similar. Overall, feline and mouse AIM were functionally equivalent in their ability to enter adipocytes and hepatocytes, and induce lipolysis.

### Accelerated obesity and fatty liver in the absence of AIM release in blood

**Figure 2:**
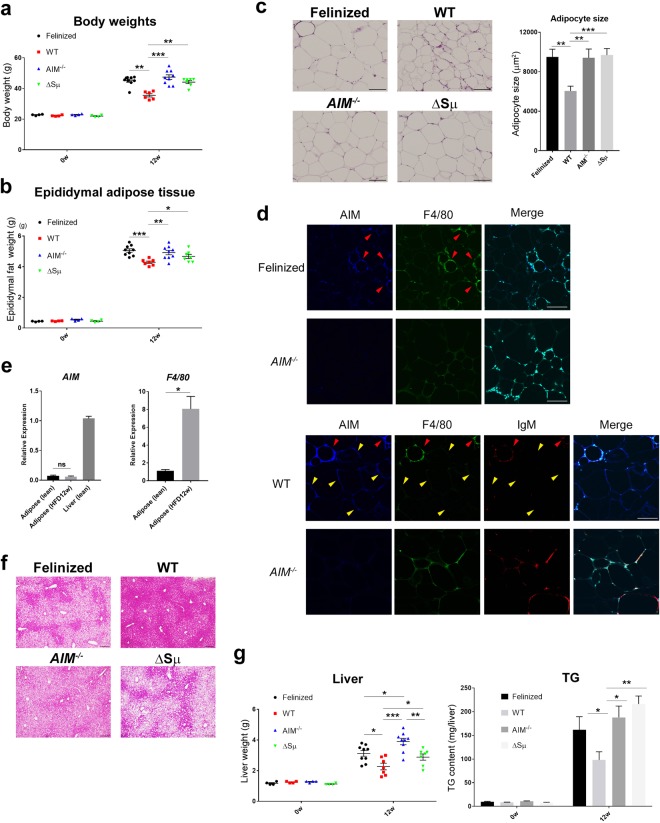
Effect of serum IgM-free AIM on obesity and liver steatosis. (**a,b)** Weights from AIM-felinized, WT, *AIM*^−/−^, and Δsµ mice before and after being fed an HFD for 12 weeks (n = 4 before HFD and n = 6–9 for 12-week HFD per group). Error bar indicates the SEM. **(a)** Body weights. **(b**) Weights of epidydimal adipose tissues. **(c)** Representative photomicrographs of epididymal fat tissues from AIM-felinized, WT, *AIM*^−/−^, and Δsµ mice fed an HFD for 12 weeks stained with H&E. Adipocyte sizes of 50 independent adipocytes in different areas were evaluated. Results are presented as average ± SEM (in μm^2^). Scale bars, 100 μm. (**d**) Representative photomicrographs of epididymal fat tissues from AIM-felinized, WT, and *AIM*^−/−^ mice (fed an HFD for 12 weeks) stained for AIM (blue), F4/80 (macrophage marker; green), and IgM (red, WT and *AIM*^−/−^ mice. See Supplementary Fig. 1 for AIM-felinized mice). Yellow arrows represent where IgM-free AIM signals exist, while red arrows indicate where AIM is co-stained with IgM. Scale bars, 100 μm. **(e)** The mRNA levels of *AIM* and *F4/80* were assessed by qPCR using RNA isolated from epididymal fat in WT mice before or after being fed an HFD for 12 weeks. Values were normalized to those of *GAPDH* and presented as the expression relative to that of *AIM* from lean WT mice liver and of *F4/80* from fat tissues before being fed an HFD (n = 4 per group). Error bar indicates the SEM. **(f)** Representative photomicrographs of liver from AIM-felinized, WT, *AIM*^−/−^, and Δsµ mice fed an HFD for 12 weeks stained with H&E. Scale bars, 100 µm. **(g)** Liver weights and TG contents from AIM-felinized, WT, *AIM*^−/−^, and Δsµ mice before and after being fed an HFD for 12 weeks (n = 4 before HFD and n = 6–9 for 12-week HFD per group). Error bar indicates the SEM.

Having observed that feline and mouse AIM were functionally comparative, we then addressed whether AIM release was needed to suppress lipid storage in adipose tissue and the liver *in vivo*. AIM-felinized mice, in which AIM does not dissociate from IgM, WT mice, and *AIM*^−/−^ mice were fed an HFD. Secreted IgM-deficient (Δsµ) mice^25^ were used as controls, in which circulating AIM is at undetectable levels due to the lack of secreted IgM required for AIM storage in the blood. Note, however, in Δsµ mice, tissue-resident macrophages such as Kupffer cells produce AIM normally as in WT mice; thus, the situation of AIM was comparable with that in AIM-felinized mice (i.e., normal AIM production by macrophages but almost no functional AIM in the blood). When fed an HFD for 12 weeks, the increase in body weight was significantly accelerated in AIM-felinized mice compared with that in WT mice (Fig. 2a). The increase was slightly milder in AIM-felinized mice than in *AIM*^−/−^ mice, but the difference was not significant (Fig. 2a). As expected, Δsµ mice exhibited a similar increase in body weight as did AIM-felinized mice (Fig. 2a). Parallel results were obtained in the weight gain of epidydimal adipose tissues (Fig. 2b). In accordance, the size of adipocytes in the epidydimal fat was also significantly enlarged in the three types of mutant mice compared with that in WT mice (Fig. 2c).

We then examined the AIM internalization into obese adipocytes by immunohistochemistry. As shown in Fig. 2d, while AIM was stained within epididymal adipocytes in WT mice (lower panels), no obvious AIM signal was detected in adipocytes in AIM-felinized mice like in *AIM*^−/−^ mice (upper panels), consistent with the hyperobese phenotype observed in AIM-felinized and *AIM*^−/−^ mice. Note that IgM was not co-stained in adipocytes in WT mice, indicating that the incorporated AIM in WT mice was the one that dissociated from IgM, namely, IgM-free AIM (Fig. 2d, indicated by yellow arrows). Interestingly, in both AIM-felinized mice (Supplementary Fig. 1, indicated by red arrows) and WT mice (Fig. 2d, lower panels, indicated by red arrows), AIM was found in F4/80-positive macrophages colocalizing with IgM, suggesting that the IgM/AIM complex might be internalized by macrophages through scavenger receptors and/or Fc receptors. The AIM stained in adipocytes was not derived from the cells, as we previously demonstrated that adipocytes do not express AIM^18,26^. Accordingly, the mRNA levels of *AIM/cd5l* did not increase in obese adipose tissue compared to lean adipose tissue, despite massive macrophage infiltration (Fig. 2e). IgM-free AIM was not obvious in the serum from WT mice fed an HFD for 12 weeks unlike in AKI mice, as assessed by immunoblotting (Supplementary Fig. 2). Thus, in obese conditions, AIM may dissociate from IgM locally near adipose tissues, whereas during AKI, it appears that AIM dissociates systemically in the blood.

Similar to adipose tissue, liver steatosis was also accelerated in AIM-felinized mice compared with that in WT mice fed an HFD for 12 weeks. Although the level of steatosis appeared histologically comparable in AIM-felinized mice and *AIM*^−/−^ mice (Fig. 2f), the triacylglycerol (TG) content in the whole liver and the weight of the liver were slightly lower in AIM-felinized mice than in *AIM*^−/−^ mice, although the difference was not statistically significant (Fig. 2g). As in the fat tissue, Δsµ mice also showed an identical phenotype in the liver (Fig. 2f,g). Thus, the fact that AIM-felinized mice and Δsμ mice show liver steatosis and triacylglycerol accumulation is indicative that the absence of available blood AIM is what is contributing to the phenotype.

### States of inflammation and fibrosis in the presence or absence of IgM-free AIM

**Figure 3:**
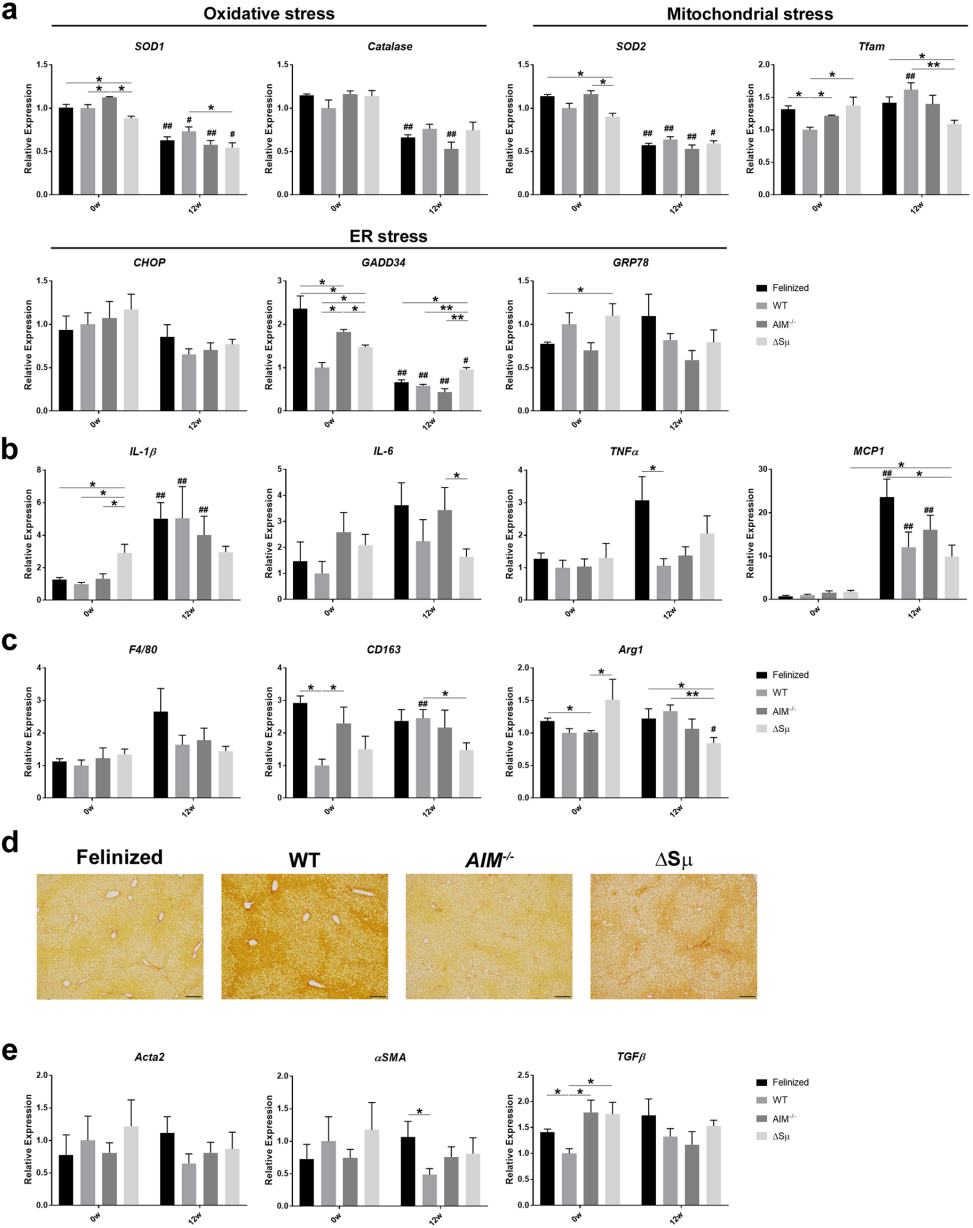
States of inflammation and fibrosis in the liver in the absence of serum IgM-free AIM. (**a**) The mRNA levels of various genes responsive to different types of stresses (i.e., endoplasmic reticulum, mitochondrial, or oxidative stress) addressed by qPCR using RNA from the whole liver of AIM-felinized, WT, *AIM*^−/−^, and Δsµ mice before and after being fed an HFD for 12 weeks (n = 4 before HFD and n = 5–6 for 12-week HFD per group). Error bar indicates the SEM. **(b,c)** The mRNA levels of inflammatory cytokine genes in the liver, as assessed by qPCR using the RNA as in (**a**). **(d**) Representative photomicrographs of liver from AIM-felinized, WT, *AIM*^−/−^, and Δsμ mice fed an HFD for 12 weeks stained with Sirius red Stain. Scale bars, 100 μm. **(e)** The mRNA levels of fibrogenetic genes in the liver, as assessed by qPCR using the same RNA as in (**a**). In **(a**–**c)** and **(e)**, *is used to represent the statistical significance between the values of each mouse strain group within the same period, whereas # is attached to the bar of 12 w when the value of 12 w was significantly changed compared with that of 0 w in the same mouse strain group.

Lipid overload in the liver often causes excess cell stress in hepatocytes, which is followed by cell death, leading to chronic liver inflammation and liver fibrosis^27,28^. Progressive inflammation and fibrosis are the most prominent risk factors for the development of HCC^29–31^. Previously, we reported that although the cell stress state in the liver was slightly enhanced in *AIM*^−/−^ mice compared with that in WT mice in line with advanced steatosis, both types of mice showed essentially similar levels of liver inflammation during liver steatosis progression^19^. As in *AIM*^−/−^ mice, AIM-felinized mice and Δsµ mice showed mild differences in the mRNA levels of some stress-responsive genes in the liver compared with those in the WT liver (Fig. 3a). Interestingly, the mRNA level of *GADD34* was significantly higher in AIM-felinized mice before the 12-week-diet (Fig. 2a). The exact reason for this observation was unclear.

The mRNA level of interleukin (IL)-1β, an inflammatory cytokine, increased significantly in WT, *AIM*^−/−^, and AIM-felinized mice to a comparable level, when fed an HFD for 12 weeks (Fig. 3b). The mRNA level of monocyte chemoattractant protein 1 (*MCP-1*) also increased significantly in those mice fed an HFD for 12 weeks, most prominently in AIM-felinized mice (Fig. 3b). The levels for other inflammatory cytokines such as *IL-6* and tumour necrosis factor alpha (*TNFα*) did not increase significantly after a 12-week-diet, but they were relatively higher in AIM-felinized mice than in other types of mice after the diet (Fig. 3b). Accordingly, the increase in *F4/80* mRNA level reflecting inflammatory macrophage recruitment in the liver was most prominent in AIM-felinized mice after the diet, but the increase after the diet was, however, not significant (Fig. 3c). The mRNA levels of M2 macrophage genes, *CD*1*63* and *Arg*-*1*, did not change significantly by HFD, except in WT mice, which showed an increase in the *CD163* level, and Δsµ mice, which showed a decrease in the *Arg*-*1* level (Fig. 3c). Thus, the change of inflammatory state brought about by HFD was not profoundly different in AIM-felinized mice compared to other types of mice, though there were subtle variations among the four genotypes. Consistent with this observation, the histological progression of liver fibrosis was largely comparable in all types of mice (Fig. 3d). This was also true with regard to the mRNA levels of fibrogenetic genes such as *Acta2*, *αSMA*, and *TGFβ* (Fig. 3e).

Based on the similar inflammatory and fibrotic states in the liver, it is likely that all types of mice were equally susceptible to HCC. We previously found that AIM induces necrotic cell death in cancerized hepatocytes via activation of complement cascades, resulting in a 0% incidence of HCC in WT mice compared with an almost 100% incidence in *AIM*^−/−^ mice fed an HFD for 1 year^19^.

### Suppressed HCC development even in the absence of IgM-free AIM

**Figure 4:**
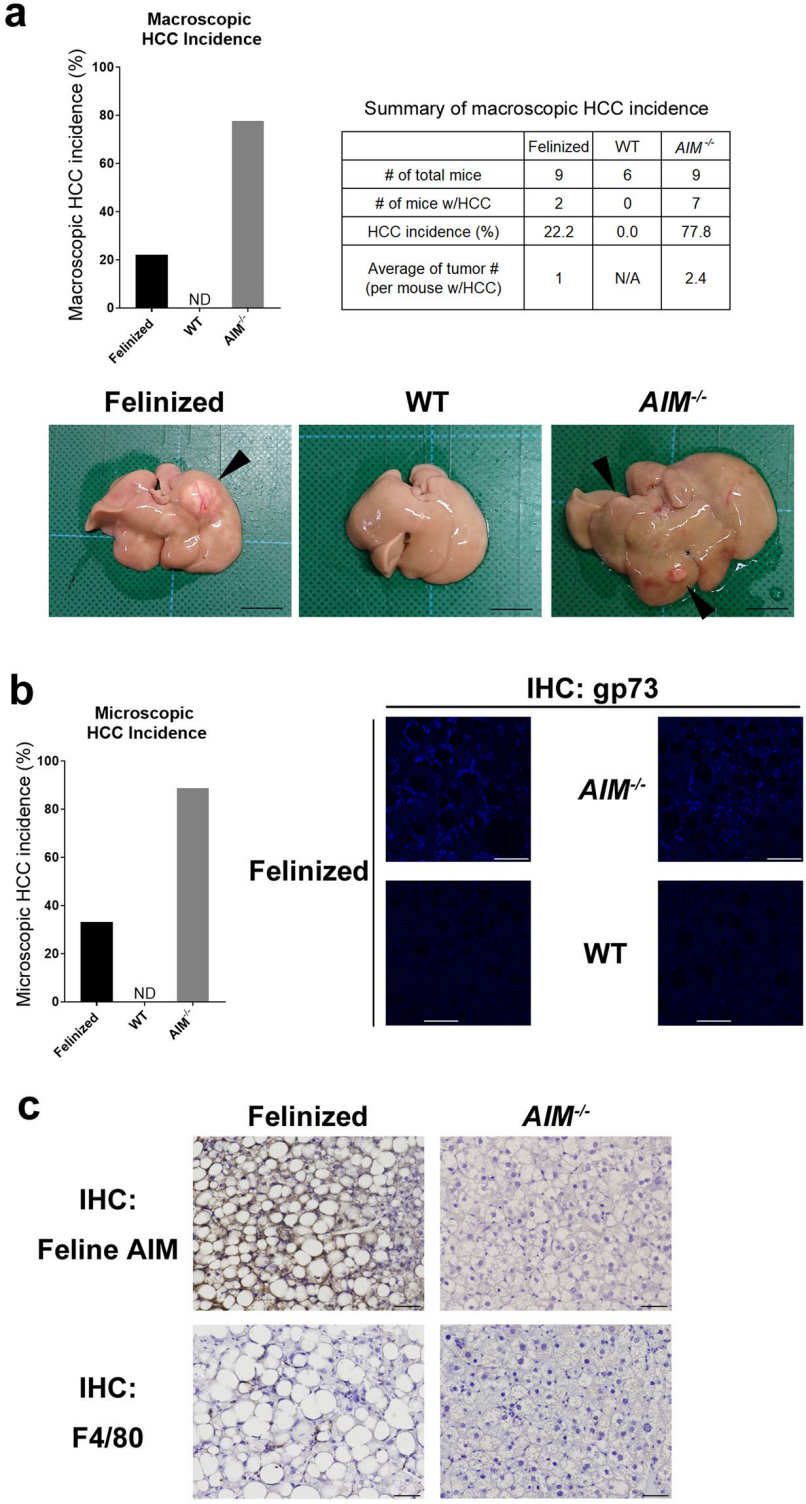
Regulation of HCC development by Kupffer cell-derived AIM. **(a)** (Graph) Macroscopic incidence of HCC in AIM-felinized, WT, and *AIM*^−/−^ mice fed an HFD for 52 weeks (n = 6 for WT; n = 9 for AIM-felinized and *AIM*^−/−^ mice per group). (Summary Box) the number of total and HCC-harbouring mice, and the average of the number of tumour. (Photos) Representative photos of the liver of three types of mice. HCC tumorus are indicated by arrows. **(b)** (Graph) Histological incidence of HCC in AIM-felinized, WT, and *AIM*^−/−^ mice fed an HFD for 52 weeks (n = 6 for WT and n = 9 for AIM-felinized and *AIM*^−/−^ mice per group). (Photos) Representative photomicrographs of H&E staining of the liver from mice fed an HFD for 52 weeks. Scale bars, 50 µm. **(c)** Representative photomicrographs of the liver showing staining for gp73 (blue). Scale bars, 50 µm. **(d)** Representative photomicrographs of region with liver tumours from AIM felinized mice and *AIM*^−/−^ mice fed an HFD for 52 weeks stained for feline AIM or F4/80. Scale bars, 50 μm.

Based on the aforementioned results, we were interested in determining whether blood AIM dissociated from the IgM pentamer was involved in HCC tumour prevention (i.e., HCC incidence in AIM-felinized mice). In accordance with our previous report, most *AIM*^−/−^ mice developed multiple liver tumours when fed an HFD for 1 year (52 weeks) (Fig. 4a). Histologic analysis revealed that the tumours exhibited typical HCC, (Fig. 4b). In contrast, WT mice did not develop HCC after the same HFD challenge (Fig. 4a,b). IgM-free AIM was detected in the serum from these obese WT mice (Supplementary Fig. 2), suggesting that AIM dissociated from the IgM pentamer, which may have contributed to HCC prevention analogous to its protective effect on lipid deposition in fat tissue and the liver. However, to our surprise, the incidence of HCC development was markedly suppressed in AIM-felinized mice both macroscopically (Fig. 4a, graph) and histologically (Fig. 4b). It is noteworthy that the number of tumour in AIM-felinized mice harbouring HCC was only one per liver section, in contrast to the multiple HCC tumours found in *AIM*^−/−^ mice (Fig. 4a, summary box and photos). The tumours developed in AIM-felinized mice and *AIM*^−/−^ mice were stained for gp73, a marker commonly used for HCC evaluation^32^ (Fig. 4c). In AIM-felinized mice harbouring HCC, immunohistochemical analysis revealed that feline AIM deposited at the tumour, accompanied by F4/80^+^ macrophage accumulation (Fig. 4d). The size was larger and lipid storage was more pronounced in HCC cells from AIM-felinized mice than from *AIM*^−/−^ mice (Fig. 4d). Thus, unlike its effects in preventing lipid storage in adipose tissue and the liver, IgM-free AIM produced in the blood may not be the major contributor in HCC prevention. Thus, it is likely that AIM secreted from liver Kupffer cells^1,17^ and recruited to cancerized hepatocytes, may directly accumulate at the cell surface, promoting necrotic cancer cell death.

## Discussion

Through its unique association with the IgM pentamer, a large amount of AIM protein (µg/mL orders) is stored in the blood in an inactivated form. During specific diseases, AIM is released from IgM on demand and facilitates disease repair^12^. Because AIM exhibits protective effects against various diseases, our current study focused on the importance of AIM release (i.e., activation of blood AIM) in the repair and/or prevention of different diseases. To this end, we employed AIM-felinized mice, in which AIM does not dissociate from IgM, due to the extremely high binding affinity of feline AIM with the IgM-Fc region. Δsµ mice are a good tool for assessing this question, as AIM is not protected from renal excretion due to the lack of secreted IgM, and as such, blood AIM is at undetectable levels. As expected, the two types of mice exhibited similar phenotypes in the context of the protective effects of AIM against different diseases. The absence of IgM-free AIM (due to no release from IgM in AIM-felinized mice, and to no storage of AIM in the blood of Δsμ mice) caused hyperobesity in mice, indicating that the regulation of lipid deposition in adipose tissue was due to circulating AIM. As we previously reported that AIM is stained in infiltrating M1 macrophages and forms crown-like structures in the adipose tissue of obese WT mice^18^, one might wonder whether AIM is produced by the infiltrating macrophages and directly contributes to lipolysis. This is not likely, however, because resident macrophages such as Kupffer cells but not bone marrow-derived macrophages (including macrophages recruited to adipose tissue) express AIM, as also evident by the lack of increase of *cd5l*/*AIM* mRNA levels in adipose tissue. Thus, AIM stained at infiltrating macrophages should be incorporated into blood AIM. Although not significant, the increase in body weight upon HFD was slightly but obviously milder in AIM-felinized and Δsµ mice than in *AIM*^−/−^ mice. As AIM production in adipose tissue (by infiltrating macrophages) appeared unlikely, it was possible that a small amount of feline AIM was released locally and incorporated into adipocytes. However, in Δsµ mice, a subtle lipolytic effect might have been caused by a small amount of blood AIM, at levels that would be undetectable by immunoblotting. Essentially the same consequence was observed in the progression of liver steatosis; that is, suppression of fatty liver also appeared to be managed by circulating AIM after its release from IgM.

The most interesting observation in this study was the discrepancy in IgM-free effects on fatty liver and HCC; HCC incidence was markedly decreased in AIM-felinized mice compared with that in *AIM*^−/−^ mice. This result strongly suggests that AIM produced by liver Kupffer macrophages might directly target cancerized hepatocytes and induce cell death without a detour through the bloodstream. Our current observation of strong deposition of AIM at the liver tumour region accompanied by marked Kupffer cell accumulation may support this idea. Interestingly, many tumours developed in AIM-felinized mice consisting of large and lipid-laden cancer cells, indicating mature HCC cells, whereas those that developed in *AIM*^−/−^ mice were smaller with less lipid deposition, indicating less mature cells. The precise reason for the morphological difference is unclear, but it is possible that mature HCC cells, which may be more resistant to cell death caused by AIM, preferentially survive and develop tumours in AIM-felinized mice. It is noteworthy that blood AIM might also contribute to HCC prevention, as HCC incidence was higher in *AIM*^−/−^ than in WT mice.

Our results suggest that an increase in AIM production by liver Kupffer cells may have therapeutic effects against HCC. Because AIM expression in tissue macrophages is transcriptionally regulated by LXR/RXR heterodimers^2–4^, LXR agonists may enhance AIM production in Kupffer cells and have therapeutic effects against HCC. Indeed, recent studies have demonstrated that LXR has beneficial functions in human HCC^33,34^. However, the molecular mechanisms underlying how LXR exerts its anti-tumour effects have been unclear. Based on our current results, it is possible that one of the mechanisms for the therapeutic effects of LXR activation in HCC might be the increase in AIM production in tissue macrophages. Additional studies confirming the decreased incidence of HCC upon administration of an LXR agonist in AIM-felinized mice are necessary. In addition, accumulating evidence has demonstrated that LXR has anticancer roles in various human cancers including prostate, breast, ovarian, skin, and colon cancers^35–39^. In some, the effect might be due to the increase in AIM production by resident macrophages in each tissue via LXR activation.

In fact, a discrepancy in the incidence of fatty liver and HCC is often observed in cats; they are susceptible to obesity and fatty liver, whereas the development of HCC is rare^40–43^. Hence, many disease phenotypes in cats can be explained by the lack of blood AIM activation. Further studies are needed to reveal the therapeutic applications of AIM for treating incurable diseases in cats.

In conclusion, our study depicted different modes of AIM effects in facilitating disease suppression, thereby giving a mechanistic insight into the therapeutic effects of LXR activation in HCC.

## Methods

### Animals

*AIM*^−/−^ mice had been backcrossed to C57BL/6 (B6) for 15 generations before they were used in our experiments. AIM-felinized mice were generated as previously reported^17^. C57BL/6 mice were purchased from CLEA Japan, Inc. (Tokyo, Japan), and ΔSµ mice were purchased from The Jackson Laboratory. All animal experiments were performed in strict accordance with recommendations from the Guide for the Care and Use of Laboratory Animals of the National Institutes of Health. The protocol was approved by the Committee on the Ethics of Animal Experiments of the University of Tokyo (Permit No. P10-143).

### Antibodies and reagents

The antibodies and reagents used for histological and biochemical experiments are as follows. Primary antibodies: AIM (rab2 rabbit polyclonal for mice AIM in western blotting and immunohistochemistry, PAC-11 rabbit polyclonal for feline AIM in western blotting, and clone #33 mouse monoclonal for feline AIM in western blotting and immunohistochemistry; established in our laboratory), IgM (A21044, goat polyclonal, Alexa 594 conjugated, Thermo Fisher Scientific, Waltham, MA), F4/80 (clone: BM8 for immunohistochemistry; Thermo Fisher Scientific), CD36 (clone: MF3 for immunohistochemistry; Abcam, Cambridge, UK) and gp73 (goat polyclonal for immunohistochemistry; Santa Cruz, Dallas, TX). Secondary Antibodies: goat anti-rabbit IgG (H + L) horseradish peroxidase (HRP) conjugated (Thermo Fisher Scientific), Alexa Fluor 488- or 594-conjugated anti-rat IgG, and Alexa Fluor 647-conjugated anti-rabbit IgG (Thermo Fisher Scientific). In addition, the Peroxidase Labeling Kit-NH2 (Dojindo Molecular Technologies, Rockville, MD) was used for conjugating HRP to clone #33, and the Histofine Simple Stain Mouse MAX-PO (rat) (Nichirei, Tokyo, Japan) and Histofine Mouse Stain Kit (Nichirei) were used.

### Production of rAIM

For mouse AIM (mAIM), HEK293T cells were transfected with the pCAGGS-mAIM plasmid and cultured in Dulbecco’s modified Eagle medium (DMEM) + GlutaMAX medium (Gibco, Gaithersburg, MD) supplemented with 5% foetal bovine serum (FBS) for 3 days. rAIM was purified from the culture supernatant using a rat anti-mouse AIM monoclonal antibody (clone #36, made in house)-conjugated HiTrap NHS-activated HP column (GE Healthcare Life Sciences, Pittsburgh, PA). Bound proteins were eluted with 0.1 M glycine-HCl (pH 2.3) and neutralized with 1 M Tris-HCl (pH 8.5). Proteins were concentrated using Amicon Ultra Filter Concentrators (Millipore, Billerica, MA) and stored at −80 °C in phosphate-buffered saline (PBS). Endotoxin levels were measured using the Limulus Color KY Test Wako (Wako Pure Chemical Industries, Ltd., Osaka, Japan). Protein concentration was determined by the bicinchoninic acid assay according to the manufacturer’s protocol (Pierce, Rockford, IL). Feline rAIM was produced by the same protocol with some modifications. Briefly, pCAGGS-feline AIM plasmids were used. A mouse anti-feline AIM monoclonal antibody (clone #33) was used for purification of feline AIM, and bound protein was eluted with 0.1 M glycine-HCl (pH 2.0).

### Cell Lines

HEK293T cells and 3T3-L1 cells were grown at 37 °C in DMEM + GlutaMax medium supplemented with 10% FBS and gentamicin (Gibco).

### Isolation of mouse primary hepatocytes

Mouse primary hepatocytes were isolated by two-step collagenase perfusion method with slight modifications^44^. Briefly, mice were anesthetized with isoflurane and liver was preperfused via portal vein with perfusion buffer (Hank’s balanced salt solution [HBSS], 0.5 mM EGTA, 25 mM HEPES); next, the liver was perfused with digestion buffer (DMEM, 100 CDU/ml type 4 collagenase, 50 μg/ml trypsin inhibitor, 15 mM HEPES). After the extraction of the liver, the cells were dispersed by blade mincing. The cells obtained were filtered through a 100 μm nylon cell strainer and centrifuged (500 *g*, 2 min). For further purification, the remaining cells were subjected to 45% Percoll density gradient centrifugation for 10 min at 1200 *g*. Cell viability measured by trypan blue dye exclusion was >95%.

### AIM incorporation assay (immunocytochemistry)

pFLAG-CMV2 vector (Sigma-Aldrich) was used to generate expression vectors for mouse CD36. Mouse and feline rAIM were labelled with FITC (Dojindo) and used for experiments. HEK293T cells were cultured in Lab-Tek II 4-well chamber slides (Nunc) 2 days before the assay, and transfected with mouse CD36 expression vector 1 day before the assay. rAIM was added to each well at 20 µg/mL and incubated for 30 min at 37 °C. Then, the cells were fixed in 4% paraformaldehyde (PFA) in PBS for 15 min at room temperature and stained for CD36. The nuclei were stained with Hoechst 33342 and mounted in Prolong Gold Antifade reagent (Thermo Fisher Scientific). Incorporation of AIM was analysed by confocal microscopy (FV10i; Olympus, Tokyo, Japan).

### AIM function assay (3T3-L1 differentiation and Oil Red O staining)

3T3-L1 cells were cultured in DMEM supplemented with 10% FBS in Lab-Tek II 4-well chamber slides 4 days before induction. Medium was changed every 2 days. 3T3-L1 cells were stimulated with insulin (1 µg/mL), dexamethasone (DEX, 1 µM), and 3-isobutyl-1-methyl xanthine (IBMX, 500 µM), and cultured for 2 days. Then cells were cultured in DMEM with insulin (1 µg/mL) for 2 days and cultured in DMEM alone for an additional 2 days. Differentiated 3T3-L1 adipocytes were challenged with rAIM (10 µg/mL) or PBS for 6 days. After rAIM treatment, cells were fixed in 4% PFA in PBS for 15 min at room temperature and stained with Oil Red O (Muto Pure Chemicals, Bunkyo, Japan). The numbers of droplet-containing cells are shown (cells/microscopic field; 440 × 330 µm, average of 5 fields). Differentiated 3T3-L1 adipocytes incubated with rAIM (20 µg/mL) or PBS for 2 days were also used for quantitative PCR (qPCR).

### qPCR assay

Quantitative evaluation of mRNA was performed by the ΔΔC_T_ method using the QuantStudio 3 Real-Time PCR system (Invitrogen, Carlsbad, CA). Sequences of the oligonucleotides used are presented in Supplementary Table 1.

### Histology

Mouse epididymal fat and liver were fixed in 4% PFA for 24 h, followed by embedding in paraffin. Hemotoxylin and eosin (H&E) staining, Sirius Red staining, and immunostaining were performed on 4 µm sections of paraffin-embedded tissue blocks. *H&E staining*: Paraffin-embedded fat and liver sections were stained with H&E. Digital images were acquired with an inverted microscope (FSX-100; Olympus). Adipocyte size was analysed with Image J and an average of 50 cells are shown. *Sirius Red staining*: Paraffin-embedded liver sections were stained with hematoxylin and then incubated with Sirius Red staining solution (0.1% Direct Red diluted in saturated picric acid) for 1 h at room temperature in the dark. Digital images were acquired with an inverted microscope (FSX-100; Olympus). *Immunohistochemistry*: 4 µm sections of PFA-fixed paraffin-embedded fats were stained for feline AIM and F4/80. The nuclei were stained with Hoechst 33342 and mounted using Prolong Gold Antifade reagent. Incorporation of AIM was analysed by confocal microscopy (FV10i; Olympus). Paraffin-embedded livers were stained for gp73. The nuclei were stained with Hoechst 33342 and analysed by confocal microscopy. Liver sections were also stained for F4/80, followed by incubation with Histofine Simple Stain Mouse MAX-PO (rat) (Nichirei) for 30 min or stained for feline AIM using the Histofine Mouse Stain Kit (Nichirei). After staining with DAB, the sections were counterstained with hematoxylin. The specimens were subjected to analysis using an inverted microscope (FSX-100; Olympus).

### Liver TG content

Approximately 30 mg liver was homogenized with 1 mL 5% NP-40 in water. Samples were heated to 80 °C on a heat block for 5 min, cooled to room temperature, and then heated one more time. After centrifugation, the supernatant was diluted and TG content was measured using the LabAssay™ Triglyceride (Wako).

### Statistical analysis

The mean values were measured from at least three replicates. A two-tailed Mann–Whitney test was used to calculate the *P* values. ***P < 0.001, **P < 0.01, *P < 0.05. or ^##^*P* < 0.01, ^#^*P* < 0.05. Error bars: standard error of the mean.

## Electronic supplementary material

Supplementary Figures
