# Supplementary material for: Independent modes of disease repair by AIM protein distinguished in AIM-felinized mice
Source: Sci Rep. 2018 Sep 3;8:13157. doi: 10.1038/s41598-018-31580-6 (PMC6120884; doi:10.1038/s41598-018-31580-6)
Supplement: Supplementary file 1 — Supplementary Figures [file 41598_2018_31580_MOESM1_ESM.docx]

**Independent modes of disease repair by AIM protein distinguished in AIM-felinized mice**

Ryoichi Sugisawa^1, 5^*, Ginga Komatsu^1^*, Emiri Hiramoto^1^, Naoki Takeda^2^, Ken-ichi Yamamura^2^, Satoko Arai^1^, Toru Miyazaki^1, 3, 4‡^

*^1^Laboratory of Molecular Biomedicine for Pathogenesis, Center for Disease Biology and Integrative Medicine, Faculty of Medicine, The University of Tokyo, Tokyo 113-0033, Japan.*

*^2^Center for Animal Resources and Development, Kumamoto University, Kumamoto 860-0811, Japan.*

*^3^CREST, Japan Agency for Medical Research and Development, Tokyo 113-0033, Japan.*

*^4^Max Planck-The University of Tokyo Center for Integrative Inflammology, Tokyo 113-0033, Japan.*

‡Correspondence should be addressed to Toru Miyazaki

Laboratory of Molecular Biomedicine for Pathogenesis, Center for Disease Biology and Integrative Medicine, Faculty of Medicine, The University of Tokyo, 7-3-1 Hongo, Bunkyo-ku, Tokyo 113-0033, Japan.

(TEL) +81-3-5841-1436 (FAX) +81-3-5841-1438 (E-mail) [tm@m.u-tokyo.ac.jp](mailto:tm@m.u-tokyo.ac.jp)

^5^Present address: Trinity Biomedical Sciences Institute, Trinity College Dublin, Dublin, Ireland

* Co-first author

**
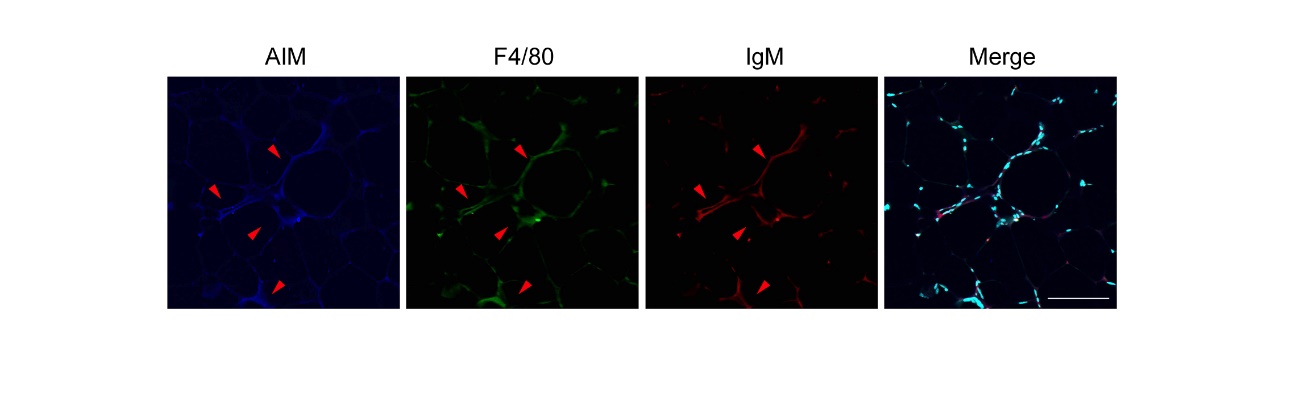
Supplementary Fig. 1. AIM and IgM were co-stained in F4/80^+^ macrophages but not in adipocytes in adipose tissue of AIM-felinized mice.** Representative photomicrographs of epididymal fat tissues from AIM-felinized mice (fed an HFD for 12 weeks) stained for AIM (blue), F4/80 (macrophage marker; green), and IgM. Red arrows; AIM is co-stained with IgM. Scale bar, 100 μm.


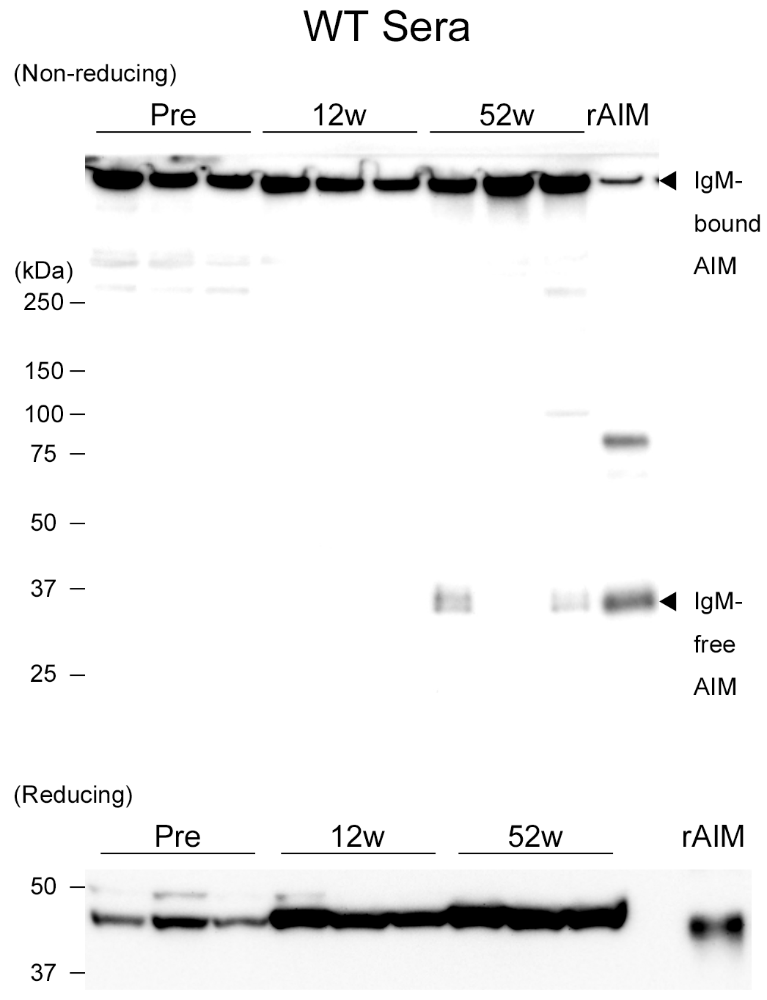


**Supplementary Fig. 2. AIM dissociation states in obese mice.**

Representative immunoblot of AIM in serum using an anti-AIM polyclonal antibody (Rab2). WT mice before and after being fed an HFD for 12 or 52 weeks. Serum (1 µL) and rAIM (20 ng) were loaded as controls.

| Name | Sequence (5’ - 3’) |
| --- | --- |
| f- GAPDH | AGAACATCATCCCTGCATCC |
| r- GAPDH | CACATTGGGGGTAGGAACAC |
| f- FSP27 | CTGGAGGAAGATGGCACAATCGTG |
| r- FSP27 | CAGCCAATAAAGTCCTGAGGGTTCA |
| f- Perilipin | TGCTGGATGGAGACCTC |
| r- Perilipin | ACCGGCTCCATGCTCCA |
| f- Adipophilin | AAGCATCGGCTACGACGACAC |
| r- Adipophilin | GGACAGTCTGGCATGTAGTCTGGA |
| f- AIM | GAGGACACATGGATGGAATGT |
| r- AIM | ACCCTTGTGTAGCACCTCCA |
| f- F4/80 | CCTGGACGAATCCTGTGAAG |
| r- F4/80 | GGTGGGACCACAGAGAGTTG |
| f- SOD1 | CAGGACCTCATTTTAATCCTCA |
| r- SOD1 | TGCCCAGGTCTCCAACAT |
| f- Catalase | CCTTCAAGTTGGTTAATGCAGA |
| r- Catalase | CAAGTTTTTGATGCCCTGGGT |
| f- SOD2 | TGCTCTAATCAGGACCCATTG |
| r- SOD2 | GTAGTAAGCGTGCTCCCACAC |
| f- Tfam | CAAAGGATGATTCGGCTCAG |
| r- Tfam | AAGCTGSSTATATGCCTGCTTTTC |
| f- CHOP | GCGACAGAGCCAGAATAACA |
| r- CHOP | GATGCACTTCCTTCTGGAACA |
| f- GADD34 | GACCCCTCCAACTCTCCTTC |
| r- GADD34 | CTTCCTCAGCCTCAGCATTC |
| f- GRP78 | GGAAAGAAGGTTACCCATGC |
| r- GRP78 | AGAAGAGACACATCGAAGGT |
| f- IL1β | CTGGTGTGTGGACGTTCCCATTA |
| r- IL1β | CCGACAGCACGAGGCTTT |
| f- IL-6 | TCTATACCACTTCACAAGTCGGA |
| r- IL-6 | GAATTGCCATTGCACAACTCTTT |
| f- TNFα | CCCTCACACTCAGATCATCTTCT |
| r- TNFα | GCTACGACGTGGGCTACAG |
| f- MCP1 | CATCCACGTGTTGGCTCA |
| r- MCP1 | GATCATCTTGCTGGTGAATGAGT |
| f- CD163 | CCTGGATCATCTGTGACAACA |
| r- CD163 | TCCACACGTCCAGAACAGTC |
| f- Arg1 | CTCCAAGCCAAAGTCCTTAGAG |
| r- Arg1 | AGGAGCTGTCATTAGGGACATC |
| f- Acta2 | CTCTCTTCCAGCCATCTTTCAT |
| r- Acta2 | TATAGGTGGTTTCGTGGATGC |
| f- αSMA | ACTCTCTTCCAGCCATCTTTCA |
| r- αSMA | ATAGGTGGTTTCGTGGATGC |
| f- TGFβ | TGGAGCAACATGTGGAACTC |
| r- TGFβ | CAGCAGCCGGTTACCAAG |

**Supplementary Table 1. Sequences of oligonucleotides used in QPCR.**
